# Clusterin in Alzheimer’s Disease: Mechanisms, Genetics, and Lessons From Other Pathologies

**DOI:** 10.3389/fnins.2019.00164

**Published:** 2019-02-28

**Authors:** Evangeline M. Foster, Adrià Dangla-Valls, Simon Lovestone, Elena M. Ribe, Noel J. Buckley

**Affiliations:** Department of Psychiatry, University of Oxford, Oxford, United Kingdom

**Keywords:** neurodegeneration, amyloid, cell death, neuroprotection, DKK1, oxidative stress, Wnt signaling

## Abstract

Clusterin (CLU) or APOJ is a multifunctional glycoprotein that has been implicated in several physiological and pathological states, including Alzheimer’s disease (AD). With a prominent extracellular chaperone function, additional roles have been discussed for clusterin, including lipid transport and immune modulation, and it is involved in pathways common to several diseases such as cell death and survival, oxidative stress, and proteotoxic stress. Although clusterin is normally a secreted protein, it has also been found intracellularly under certain stress conditions. Multiple hypotheses have been proposed regarding the origin of intracellular clusterin, including specific biogenic processes leading to alternative transcripts and protein isoforms, but these lines of research are incomplete and contradictory. Current consensus is that intracellular clusterin is most likely to have exited the secretory pathway at some point or to have re-entered the cell after secretion. Clusterin’s relationship with amyloid beta (Aβ) has been of great interest to the AD field, including clusterin’s apparent role in altering Aβ aggregation and/or clearance. Additionally, clusterin has been more recently identified as a mediator of Aβ toxicity, as evidenced by the neuroprotective effect of *CLU* knockdown and knockout in rodent and human iPSC-derived neurons. *CLU* is also the third most significant genetic risk factor for late onset AD and several variants have been identified in *CLU*. Although the exact contribution of these variants to altered AD risk is unclear, some have been linked to altered *CLU* expression at both mRNA and protein levels, altered cognitive and memory function, and altered brain structure. The apparent complexity of clusterin’s biogenesis, the lack of clarity over the origin of the intracellular clusterin species, and the number of pathophysiological functions attributed to clusterin have all contributed to the challenge of understanding the role of clusterin in AD pathophysiology. Here, we highlight clusterin’s relevance to AD by discussing the evidence linking clusterin to AD, as well as drawing parallels on how the role of clusterin in other diseases and pathways may help us understand its biological function(s) in association with AD.

## Introduction

Alzheimer’s disease (AD) is the most common form of dementia, accounting for over 60% of the 46.8 million cases worldwide. Due to an increasing aging population, this number is predicted to rise to over 130 million cases by 2050 (Prince et al., 2015; Alzheimer Association, 2016). Currently, there are no treatments that prevent or slow the progression of AD, and this is in part explained by the lack of mechanistic understanding of the processes underlying the disease. Although the etiology is unknown, AD is considered a multifactorial disease with age, lifestyle, and genetics as main contributing factors.

Mutations in genes such as *PSEN1*, *PSEN2*, and *APP* result in the rare, familial, early onset forms of AD, while over 20 genes have been identified that influence the risk of the more common, sporadic, late onset AD (LOAD) (Van Cauwenberghe et al., 2016). In 2009, two large independent Genome Wide Association Studies (GWAS) identified clusterin (*CLU)* as a novel LOAD-risk gene (Harold et al., 2009; Lambert et al., 2009) and numerous single nucleotide polymorphisms (SNPs) were identified as susceptibility loci in these and subsequent studies (Seshadri et al., 2010; Tan et al., 2016). *CLU* is now considered the third greatest genetic risk factor for LOAD, after *APOE* and *BIN1.* From histopathological to biomarker studies, numerous lines of evidence also suggest a link between clusterin and AD, such as the observation that clusterin is upregulated in the hippocampus and cortex of the AD brain, colocalizing with amyloid beta (Aβ) plaques (May et al., 1990). Or later, it was demonstrated that clusterin is upregulated in AD cerebrospinal fluid (CSF) (Nilselid et al., 2006). Recently, CSF clusterin levels were used in an endophenotype-based approach to try to identify novel loci that might be linked to AD pathogenesis through an alteration of clusterin in CSF (Deming et al., 2016). Additionally, higher plasma clusterin levels have been associated with increased hippocampal atrophy and increased rate of clinical progression (Thambisetty et al., 2010, 2011), suggestive of clusterin as a promising biomarker. However, although a multitude of genetic, biomarker, and *post-mortem* evidence suggests a role for clusterin in AD, it is unclear as to whether clusterin is a causal factor leading to AD development or is a contributing factor to disease progression. Either way, it is important to identify clusterin’s mechanism of action. We anticipate that the groundswell of CRISPR-based studies aimed at introducing and correcting specific variants will be pivotal in this regard.

Clusterin was traditionally referred to as an extracellular chaperone (Humphreys et al., 1999; reviewed in Satapathy, 2017) and a number of binding partners have been identified. Clusterin’s ability to interact and bind to Aβ appears to alter aggregation and promote Aβ clearance, suggesting a neuroprotective role (DeMattos et al., 2004; Bell et al., 2007; Nuutinen et al., 2007; Yerbury and Wilson, 2010; Cascella et al., 2013; Narayan et al., 2014; Merino-Zamorano et al., 2016; Yeh et al., 2016; Zandl-Lang et al., 2017). However, other studies show that clusterin may in fact reduce the clearance of Aβ (Oda et al., 1995; Lambert et al., 1998; DeMattos et al., 2002; Nielsen et al., 2010; Mulder et al., 2014) and may be a key mediator regulating Aβ-induced neurotoxicity (Killick et al., 2014; Robbins et al., 2018). Finally, it has been argued that the nature of the interaction between Aβ and clusterin is dependent on the clusterin:Aβ ratio (Yerbury et al., 2007) and the factor in excess might determine whether clusterin exhibits neuroprotective or neurotoxic properties.

As can be readily appreciated, many previous attempts have been made to understand the contribution that clusterin plays in a number of diseases including AD (Nuutinen et al., 2009; Bertram and Tanzi, 2010; Li et al., 2014; Rohne et al., 2016), and yet this role has not been fully elucidated. In this review, we discuss the complexity of clusterin and the importance of this protein in the context of neurodegenerative diseases while drawing parallels from other fields, particularly, oncology. We discuss the different roles played by clusterin in other diseases and how these may enable us to better understand the role of clusterin in neurodegeneration and AD.

## Clusterin Complexity: From Gene to Protein

Clusterin is a ubiquitously and constitutively expressed protein found in a wide range of tissues and bodily fluids (De Silva et al., 1990; Rizzi et al., 2009). Its wide expression is accompanied by a number of attributed functions including inhibition of the complement system (Murphy et al., 1988; Jenne and Tschopp, 1989), chaperone function (Humphreys et al., 1999), lipid transport (Wang and Eckel, 2014), and regulation of cell survival and cell death pathways (Scaltriti et al., 2004a,b; Zhang et al., 2005; Takase et al., 2008; Trougakos et al., 2009; Kim et al., 2012).

Originally, the 85 kDa protein isolated from ram rete testis fluid with an aggregating or ‘clustering’ effect on Sertoli cells was identified as clusterin protein (Blaschuk et al., 1983). Over the years, however, clusterin has been re-identified numerous times and given several names based on its location of identification and function, including: testosterone repressed prostate messenger-2 (TRPM-2) (Leger et al., 1987), serum protein-40,40 (SP-40,40) (Murphy et al., 1988), complement cytolysis inhibitor (CLI) (Jenne and Tschopp, 1989), sulfated glycoprotein 2 (SGP-2) (Purrello et al., 1991), and apolipoprotein J (APOJ) (De Silva et al., 1990; James et al., 1991). It was eventually determined that all these proteins were in fact produced from the same gene (Wong et al., 1993) and the name CLU was decided on at the Workshop on Clusterin held in Cambridge in 1992 (Fritz and Murphy, 1993). Despite extensive links between clusterin and both physiological and pathological processes, the exact role of this protein is unclear. One fundamental reason for this is the complexity of *CLU* and the lack of clarity in its mRNA and protein structures.

### *CLU* Gene Structure and Regulation

**FIGURE 1:**
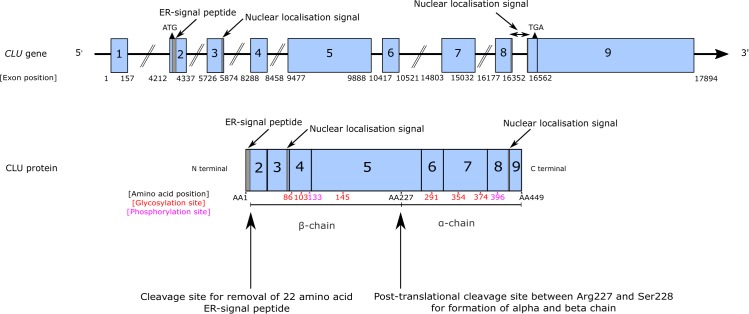
*CLU* gene and protein structure. *CLU* is a single copy gene containing nine exons. Exon 1 is a non-coding exon and two translational start sites have been identified, located in exons 2 and 3. An additional ATG has been predicted to be found in exon 1 of alternative variants of *CLU*, but their biological relevance is unclear (Prochnow et al., 2013). The main *CLU* mRNA transcript is transcript NM_001831.3 and typically translation begins at the translational start site located in exon 2. This produces an immature preproprotein (NP_001822.3) that contains exons 2–8 and the coding portion of exon 9 and includes an endoplasmic reticulum (ER)-signal peptide located in exon 2 that enables this immature protein to be processed, modified and cleaved in the traditional secretory pathway to produce mature sCLU. During the production of mature sCLU, the ER-signal peptide is cleaved and removed, and an additional cleavage event takes place between amino acid residues 227 and 228 resulting in the formation of an α-chain and a β-chain linked by disulphide bonds. Glycosylation occurs at 6 sites (indicated in red) on both the β-chain (sites 86, 103, and 145) and the α-chain (sites 291, 354, and 374). Two phosphorylation sites are also known (indicated in pink) at residues 133 and 396. Due to discrepancies in the literature, positioning of the α-chain and a β-chain, amino acid annotations, and length of the ER-signal peptide have been drawn based on current annotations provided by NCBI for the clusterin preproprotein NP_001822.3.

*CLU* is a single copy gene located at the p21-p12 locus on chromosome 8. *CLU* encodes nine exons and the majority of clusterin protein is produced from the *CLU* mRNA transcript NM_001831.3 (Prochnow et al., 2013). Translation is initiated at the start site located in exon 2. Although alternative start codons have been described in exons 1, 2, and 3 (Reddy et al., 1996; Rizzi and Bettuzzi, 2010; Prochnow et al., 2013), their functional importance has not been shown (Reddy et al., 1996; Yang et al., 2000; Leskov et al., 2003; Prochnow et al., 2013). The N-terminal endoplasmic reticulum (ER)-signal peptide located within exon 2 ensures the production of secreted protein (Wong et al., 1993), and two nuclear localization signals (NLS) are located in exon 3 and exons 8–9 (Leskov et al., 2003) (Figure 1).

Although the regulation of *CLU* is complex and incompletely characterized, it is apparent that there is a need for tight control of *CLU* expression. siRNA-knockdown of *CLU* in cancer cells increases apoptosis (Trougakos and Gonos, 2006) while overexpression of *CLU* in L929 cells potentiates the toxicity induced by TGF-β while protecting the same cells from TNF-α cytotoxicity (Humphreys et al., 1997). *CLU* mRNA was first observed to increase in the rat ventral prostate after castration, an observation initially attributed to androgen repression but is now thought to be due to castration-induced apoptosis (Montpetit et al., 1986; Leger et al., 1987). Nevertheless, *CLU* intron 1 does contain putative androgen response elements and treatment with androgens increases both clusterin mRNA and protein expression via the androgen receptor (AR) (Cochrane et al., 2007). A number of factors have now been observed to regulate *CLU* expression including NF-κB, growth factors, lipopolysaccharide, and several apoptosis-inducing agents such as ionizing radiation and oxidative stress (Saura et al., 2003; Trougakos et al., 2009; Zoubeidi and Gleave, 2012).

The *CLU* promoter is highly conserved in mammals (Steve Jones and Jomary, 2002) and contains a number of regulatory elements that may contribute to the control of *CLU* expression. These elements include activator protein-1 (AP-1), activator protein-2 (AP-2), specificity protein 1 (SP-1) motifs (Wong et al., 1993), androgen response elements (AREs) (Cochrane et al., 2007), and cyclic-AMP response elements (CREs) (Rosemblit and Chen, 1994). Cell death and apoptotic signals also regulate *CLU*. *CLU* mRNA expression was observed to be upregulated in rat hippocampus after both neocortical and hippocampal lesioning (May et al., 1990) and after ischaemia (May et al., 1992). Ionizing radiation increases *CLU* promoter activity in cultured cancer cells, an effect mediated via EGR-1 and EGR-1 consensus sites (Criswell et al., 2005). Apoptotic activator p53 represses *CLU* promoter activity and transcription in the MCF-7 breast cancer and the HCT 116 colon cancer cell lines, resulting in reduced levels of secreted clusterin (Criswell et al., 2003). The increase in *CLU* expression combined with clusterin’s extracellular chaperone activity has resulted in comparisons being made between heat shock proteins and clusterin (Poon et al., 2000; Wilson and Easterbrook-Smith, 2000; Nizard et al., 2007). The *CLU* promoter contains a 14 bp clusterin element (CLE), which differs from the heat shock element (HSE) by only a single base pair (Michel et al., 1997). During stress, this element becomes bound by HSF-1 (Michel et al., 1997) and by HSF-2 during proteasome inhibition (Loison et al., 2006), which causes an induction of *CLU* expression (Balantinou et al., 2009). Clusterin enhances HSF-1-mediated transcriptional activity and suppresses stress-induced apoptosis (Lamoureux et al., 2011). Heat shock conditions have been shown to not influence *CLU* expression in mouse astrocytes or motor neurons (Zinkie et al., 2013), suggesting the influence of heat shock signals on *CLU* expression may be cell- and/or tissue-dependent.

*CLU* is highly influenced by stress; the AP-1 binding site responds to several stress-related transcription factors including TGF-β (Herault et al., 1992; Wong et al., 1993; Jin and Howe, 1997), which reduces *CLU* expression (Jin and Howe, 1997) through the induction of *c-fos* (Marti et al., 1994; Jin and Howe, 1997, 1999; Itahana et al., 2007). TGF-β also down-regulates *CLU* in a number of cell types including porcine smooth muscle cells (Thomas-Salgar and Millis, 1994) and in rat astrocyte monotypic cultures (Morgan et al., 1995). Tissue-specific regulation has been reported in the case of TGF-β; an upregulation of *CLU* is in fact observed in epithelial and endothelial cells through the AP-1 binding site (Jin and Howe, 1997). *CLU* upregulation by TGF-β is also observed in rat astrocytes when co-cultured with microglia and oligodendrocytes (Laping et al., 1994; Morgan et al., 1995). In contrast, in fibroblasts, TGF-β1 downregulates *CLU* expression (Peix et al., 2018). Stress-activated transcription factor Y box binding protein 1 (YB-1) binds to the *CLU* promoter resulting in an upregulation of *CLU* expression (Shiota et al., 2011). Overexpression of *CLU* or YB-1 results in increased resistance of cancer cells to taxane, a drug used in the treatment of prostate cancer. Taxane, like many anti-cancer drugs, works by promoting stress-induced apoptosis, but this is reduced in the presence of elevated clusterin levels. These observations suggest that this upregulation of *CLU* during cellular stress is a protective mechanism, enabling cells to survive and adapt to stress through anti-apoptotic pathways. A variety of signals have been shown to induce *CLU* expression during stress, but downstream pathways influenced by this induction have not been fully characterized. Stress-induced rise in clusterin is predicted to be protective considering the chaperone function of clusterin and its comparisons to heat shock proteins.

*CLU* also appears to be controlled epigenetically. Several CpG islands have been identified in the *CLU* promoter (Rosemblit and Chen, 1994). Aging affects both DNA methylation and histone acetylation status, and epigenetic regulation may have an important role in *CLU* expression during aging (Gemenetzi and Lotery, 2014). *CLU* expression appears highly influenced by epigenetic factors in retinal pigment epithelial cells where valproic acid, a histone deacetylase (HDAC) inhibitor, induces a significant increase in clusterin protein expression and secretion (Suuronen et al., 2007). Suuronen et al. (2007) observed an increase in both clusterin mRNA and protein expression after treatment of retinal cells with the HDAC inhibitor, Trichostatin A. Additionally, clusterin secretion was increased after the cells were treated HDAC and DNA methyltransferase inhibitors. Hepatitis delta virus increases *CLU* expression by histone acetylation in human carcinoma cells (Liao et al., 2009). In comparison, histone deacetylation of the *CLU* promoter and histone methylation via the histone methyltransferase EZH2 in tumor cells results in *CLU* silencing (Hellebrekers et al., 2007; Wang et al., 2012). DNA demethylation by 5-aza-2′-deoxycytidine increases expression of CLU in prostate cancer cell lines (Rauhala et al., 2008). In human colon cancer cell lines, *CLU* is regulated predominantly by histone modifications such as histone 3 lysine 9 trimethylation (H3K9me3) and histone 3 lysine 4 trimethylation (H3K4me3) (Deb et al., 2015). Both H3K9me3 and H3K4me3 enrichment may result in altered expression of clusterin in the nucleus of colon cells (Deb et al., 2015). Furthermore, treatment of human astrocytes with valproic acid and the anti-cancer/HDAC inhibitor Vorinostat, resulted in the induction of *CLU* expression and an increase in clusterin secretion under therapeutic conditions in both cases (Nuutinen et al., 2010). All these observations indicate that the regulation of *CLU* is cell- and tissue-specific, and that this regulation is complex, involving a diverse array of intracellular and extracellular signals.

### Secreted Clusterin (sCLU) – Biogenesis

Mature, secreted clusterin or sCLU is derived from mRNA transcript NM_001831.3, containing exons 1–9 (Figure 1). The ATG located in exon 2 is used for the majority of clusterin translation (Prochnow et al., 2013) and results in the synthesis of the preproprotein (NP_001822.3), which is targeted to the ER and subsequently undergoes extensive post-translational modifications (reviewed in Rohne et al., 2016). Cleavage of the N-terminal ER-signal peptide within the ER produces an immature proprotein of 50 kDa, which is then modified by phosphorylation and glycosylation in the ER and the Golgi (Urban et al., 1987; Wong et al., 1993; Kapron et al., 1997; Lakins et al., 1998; Yang et al., 2000; Sabatte et al., 2011; Rohne et al., 2014), as indicated in Figure 1. Within the Golgi, cleavage between residues 227 and 228 results in the formation of the α- and β-chains linked by five disulphide bonds (Urban et al., 1987; Wong et al., 1993; Kapron et al., 1997; Yang et al., 2000). The resulting secreted protein is a highly glycosylated heterodimer (MW 75–80 kDa) consisting of two chains of 40 kDa each (Jenne and Tschopp, 1989; Kirszbaum et al., 1992; Choi-Miura and Oda, 1996) (Figure 1).

### Intracellular Clusterin – Biogenesis

Previously, clusterin was considered to be essentially a secreted protein, but subsequently intracellular clusterin species have also been described (Reddy et al., 1996; Yang et al., 2000; Nizard et al., 2007). Unlike sCLU’s biogenesis, the origin of intracellular clusterin is not well characterized and is still debated. Initially, intracellular clusterin was thought to arise from use of alternative exon 1s and splicing of *CLU* mRNA (Wong et al., 1993; Reddy et al., 1996; Leskov et al., 2003; Rizzi et al., 2009). However, the reports of multiple *CLU* mRNA transcripts (outlined in Table 1) were interpreted as a differential transcriptomic origin of intracellular and secreted clusterin proteins (Reddy et al., 1996; Schepeler et al., 2007). A variety of transcripts have been described, but they are not consistently observed in different cell types and/or are only expressed in cells after stress and at very low abundance (Reddy et al., 1996; Leskov et al., 2003; Rizzi et al., 2009; Prochnow et al., 2013), calling into question their physiological relevance in cells. The current consensus is that transcript NM_001831.3 is translated to produce the majority of clusterin protein and that non-secreted clusterin isoforms produced from other transcripts are rare (Prochnow et al., 2013; Rohne et al., 2016).

**Table 1:** Summary of described *CLU* transcripts and relevant notes regarding their discovery, annotation, and expression.

Transcript	Notes
NM_001831.3^∗^	• Main *CLU* transcript, registered in RefSeq to encode the functional secreted protein. • According to Rizzi and Bettuzzi (2010), its specific exon 1 (1a) contains a predicted functional ATG and computational prediction indicates cytoplasmic/nuclear localization, but these predictions have not been verified and are in contradiction with some reports, e.g., in Ling et al. (2012), where it was referred to as *CLU1* and its overexpression in SH-SY5Y cells produced secreted clusterin.
NM_001831 with shorter 5’-ends	• Transcripts with a completely aligned sequence to RefSeq’s NM_001831 but possessing shorter 5’-ends. • Not containing an ATG and TATA element, which is present in the extended exon 1 of NM_001831 sequence. • Observed in a human testes cDNA library and registered in GenBank as M64722.1 (Wong et al., 1994), in colorectal tissue and cell lines and termed CLU34 (Andersen et al., 2007; Schepeler et al., 2007) and in HEK-293, PC-3, MCF-7, and CaCo-2 cell lines and termed BC010514.1 due to its higher similarity with the sequence mRNA database entry BC010514.1 (Prochnow et al., 2013), and considered by these studies to be the main *CLU* transcript encoding the functional protein.
NM_001831 lacking exon 2 (known as “nuclear clusterin”, nCLU)	• Transcript attributed to alternative splicing, in which exons 1 and 3 are spliced together and exon 2 is omitted (Leskov et al., 2003). As a consequence, the ER-signal sequence is not present in the transcript and the first ATG is placed in exon 3 (in-frame with the ATG in exon 2). • Described in irradiated breast cancer MFC-7 cells (Leskov et al., 2003) and in very low amounts in HEK-293, PC-3, MCF-7, and CaCo-2 cell lines (Prochnow et al., 2013), but it has not been detected in other tissues (Andersen et al., 2007; Szymanski et al., 2011). • There is no consensus regarding the localization of the protein isoform translated from this transcript (Rohne et al., 2016): it was originally described to translocate from the cytoplasm to the nucleus (Leskov et al., 2003), but others have reported a cytoplasmic localization when a cDNA for this transcript has been overexpressed in different cell lines (Prochnow et al., 2013; Wei et al., 2009).
NR_038335.1^∗,^^∗∗^ (previously NM_203339.3)	• Also termed CLU35 (Andersen et al., 2007) and *CLU2* (Ling et al., 2012) and described to contain a unique exon 1 (1b).
NR_045494.1^∗,^^∗∗^ (previously NM_001171138.1)	• Originally termed CLU36 (Andersen et al., 2007) and later referred to as Isoform 11036 (Rizzi and Bettuzzi, 2010). • Identified by Andersen et al. (2007) through inspection of alternative splicing databases (Lee et al., 2003), predicted to localize in the nucleus (Nakai and Horton, 1999), and described to contain a unique exon 1 (1c) and share exons 2–9 with the other described transcripts at the time (NM_203339 and NM_001831). • Reported to contain a potential in-frame ATG in its exon 1 (Prochnow et al., 2013).
CR617497	• Transcript lacking exons 1, 3, and 4, found in brain tissue (Szymanski et al., 2011).


^∗^Transcript code from a currently accepted RefSeq entry (O’Leary et al., 2016)under Gene ID: 1191 (updated on September 9, 2018). ^∗∗^According to RefSeq (O’Leary et al., 2016), “This variant is represented as non-coding due to the presence of an upstream ORF that is predicted to interfere with translation of the longest ORF; translation of the upstream ORF renders the transcript a candidate for non-sense-mediated mRNA decay (NMD).”

The loss of exon 2 and absence of the ER-signal peptide is predicted to produce a single-chain intracellular clusterin that does not undergo cleavage or glycosylation (Reddy et al., 1996; Yang et al., 2000; Leskov et al., 2003; Moretti et al., 2007; Nizard et al., 2007; Trougakos et al., 2009). However, the seminal work on this line of research only demonstrated that exogenous expression of a low-abundance transcript lacking exon 2 led to cytosolic localization of a GFP-fusion protein, which was also found in the nuclei of apoptotic cells (Leskov et al., 2003). Although endogenous expression of this protein was observed to increase after treatment of cells with ionizing radiation in MCF-7 cells (Yang et al., 2000), its transcriptional origin has never been investigated, and others have not observed any nuclear localization in other cell types (Prochnow et al., 2013). Additionally, there is little evidence for the existence of a specific precursor protein in the synthesis of intracellular clusterin. The ∼49 kDa protein described by Leskov et al. (2003), which was detected in stressed cells and predicted to be a nCLU precursor, may in fact be a modified version of secreted clusterin with altered subcellular localization, given that deglycosylated, mature, secreted clusterin has a molecular weight of around ∼50 kDa (Stewart et al., 2007).

There have been numerous reports of glycosylated clusterin with altered cellular localization, particularly subsequent to induction of cellular stressors, including treatment with Nerve Growth Factor (O’Sullivan et al., 2003) and ER stress (Nizard et al., 2007; Li et al., 2013; Gregory et al., 2017). ER stress resulted in the cytoplasmic accumulation of clusterin in a *Drosophila* model of amyotrophic lateral sclerosis (ALS), reducing accumulation of TDP-43 protein inclusions and partially rescuing the ALS-like phenotype (Gregory et al., 2017). Treatment with autophagy-inducing stressors in cancer cell lines resulted in the upregulation of both clusterin mRNA and protein, which co-localized with LC3 puncta on autophagosomes’ membrane, promoting their biogenesis and increasing cell survival (Zhang F. et al., 2014). Multiple strands of evidence suggest that intracellular clusterin is a modified form of secreted clusterin. There are several ways in which this could occur, including: (i) the impaired secretion of clusterin after cellular stress resulting in the intracellular accumulation of mature, secreted clusterin; (ii) reuptake of secreted, mature clusterin after release from cells (Ghiso et al., 1993; Zlokovic et al., 1996; Kang et al., 2005); and (iii) improper trafficking of clusterin through the secretory pathway and premature escape resulting in the intracellular accumulation of incompletely modified or unglycosylated clusterin (Prochnow et al., 2013). Furthermore, nuclear compartmentalization of clusterin is increased after the direct inhibition of clusterin secretion (Sansanwal et al., 2015) and rare AD-mutations found in *CLU* have been shown to alter clusterin trafficking, resulting in intracellular accumulation and a loss of secreted clusterin (Bettens et al., 2015). Additionally, our group also observed that treatment of rodent primary neurons with Aβ leads to reduced clusterin secretion and increased intracellular clusterin (Killick et al., 2014). Collectively, these studies indicate a tight relationship between intracellular and secreted clusterin and highlight a potential role of altered clusterin trafficking in AD pathogenesis.

When considering the brain, it is important to note that astrocytes are the main source of clusterin, although subpopulations of neurons also do express clusterin (Pasinetti et al., 1994; Morgan et al., 1995). In the healthy brain, astrocytes synthesize and release clusterin into the extracellular space. After injury, in inflammatory states, and in neurodegenerative diseases like AD, both neuronal and astrocytic *CLU* expression is increased (Johnson et al., 1992; Zoli et al., 1993; Liu et al., 1999; Wiggins et al., 2003), although whether this rise is protective or toxic is unknown. It will be important for AD research to identify the source of neuronal intracellular clusterin. As we will discuss later, sCLU plays a role in cell survival pathways, a role which requires it to interact with intracellular proteins such as Ku70. Earlier studies have suggested that clusterin is taken up by cells subsequent to release (Ghiso et al., 1993; Zlokovic et al., 1996; Kang et al., 2005), a process that would enable sCLU to participate in intracellular pathways. This process may be enhanced in cells during damage or stress, as a protective mechanism against cell death (Pasinetti et al., 1994; Morgan et al., 1995).

## Clusterin in Health and Disease

The main focus of this review is to explore the role of clusterin in AD. However, understanding the role of clusterin in other pathologies, tissues, and cells lends further insight into clusterin mechanism of action that may further illuminate its role in AD. Here, we focus on clusterin’s role in other neurological disorders, cancer, and cardiovascular diseases.

### Clusterin and Neurological Disorders

Increased levels of clusterin have been observed not only in the AD brain but also in other neurodegenerative diseases, including ALS (Grewal et al., 1999), multiple sclerosis (Ingram et al., 2014), transmissible spongiform encephalopathies (Sasaki et al., 2002a), and Huntington’s disease (Labadorf et al., 2015). Similarly to the AD pathology, where clusterin co-localizes with Aβ in the senile plaques (reviewed in section “Clusterin in Aβ Metabolism”), in the case of alpha-synucleinopathies, clusterin co-localizes with cortical Lewy bodies (LBs) (Sasaki et al., 2002b). Interestingly, cortical LBs with a stronger clusterin immunostaining show a reduction in alpha-synuclein, perhaps indicating a role for clusterin in modifying its aggregation. This is in line with the well-known function of clusterin as an extracellular chaperone (Humphreys et al., 1999), although the different locations of the intracellular LBs and of mature, secreted clusterin pose the questions of how secreted clusterin could associate with LBs inside the cell, or whether clusterin could abandon the secretory pathway and, if that was the case, whether non-secreted clusterin species would still retain a chaperone function. Nevertheless, given that protein aggregation is a common pathological hallmark across neurodegenerative diseases, it is not surprising that the chaperone function of CLU has been studied in several proteinopathies. A recent study using N2a mouse neuroblastoma cultures and *in vivo Drosophila* models of ALS showed that CLU overexpression reduces TDP-43 protein aggregation and toxicity (Gregory et al., 2017). These findings were replicated in the same study using *Drosophila* photoreceptor neurons expressing pathogenic Huntingtin-Q128 and mutant (R406W) human tau proteins, where co-expression of CLU protected cells from proteotoxicity. Importantly, clusterin-mediated neuroprotection was only observed under ER stress conditions, which had previously been shown to induce clusterin translocation from the ER to the cytosol (Nizard et al., 2007; Li et al., 2013). However, once again the origin of clusterin interacting with intracellular protein aggregates remains unsolved and, if clusterin translocation from the ER occurred in this context, then it is uncertain whether the chaperone function of mature secreted clusterin would be present in these non-secreted forms.

Clusterin is also upregulated in some neurodevelopmental disorders that are not associated with proteotoxicity, such as schizophrenia and Rett syndrome. In schizophrenia, increased *CLU* mRNA levels are found in cortical neuronal subpopulations (Pietersen et al., 2014a,b), and sCLU is upregulated in prefrontal cortex homogenates (Athanas et al., 2015). The authors hypothesize that clusterin could be playing a neuroprotective role against oxidative stress mediated by TGF-β-signaling (Athanas et al., 2015), which is associated with schizophrenia pathophysiology (Bitanihirwe and Woo, 2011). *CLU* is also upregulated in the frontal cortex of Rett syndrome (RTT), a neurodevelopmental disorder mainly caused by mutations in the methyl CpG binding protein 2 (*MECP2*) gene (Gibson et al., 2010). *MECP2* siRNA knockdown led to *CLU* upregulation and furthermore, a direct interaction between MECP2 and the *CLU* promoter was shown, indicating that loss-of-function mutations in *MECP2* might lead to the overexpression of *CLU* (Gibson et al., 2010). It is worth noting that links between RTT and oxidative stress have also been established, with oxidative stress measurements correlating with pathogenicity of *MECP2* mutations and clinical severity of the disease (Leoncini et al., 2011; Filosa et al., 2015). Therefore, the reported *CLU* upregulation in RTT could again be a stress response partially driven by oxidative stress.

Hypoxia-ischaemia brain insult also triggers *CLU* expression (Zoli et al., 1993; Yasuhara et al., 1994; Walton et al., 1996; Kurian and McGuone, 2012). Some studies found a protective effect of CLU against hypoxia-ischaemia and showed that recovery from middle cerebral artery occlusion (MCAO) was better in wild-type (WT) than in *CLU*-KO mice, which had a larger inflammatory response around the scar area (Imhof et al., 2006; Wehrli et al., 2001), while overexpression of human *CLU* reduced the presence of inflammatory cells and cell death (Wehrli et al., 2001). However, clusterin’s role in hypoxia-ischaemia is debated, as some studies report a detrimental function of clusterin. In a neonatal hypoxia-ischaemia mouse model, in which tissue loss was accompanied by accumulation of clusterin in dying neurons, *CLU*-KO animals had reduced brain damage in an allele-dose-dependent manner (Han et al., 2001). At the cellular level, addition of exogenous clusterin potentiated neuronal death in mixed cultures of primary cortical neurons and astrocytes in an oxygen/glucose deprivation environment, while having no effect under normoxic conditions (Han et al., 2001). Similarly, *CLU*-KO mouse hippocampal slices showed higher tolerance to oxygen/glucose deprivation compared to WT brain slices, and the addition of clusterin increased their sensitivity to that of WT (Hakkoum et al., 2008). At this point it is not clear what determines the neuroprotective or neurodegenerative role of clusterin in hypoxia-ischaemia conditions, since there are numerous confounds in the various experimental systems including differences in the animal models, their developmental stage, over-expression versus gene KO and the measurements used to assess ischaemic brain damage (Holtzman et al., 2001; Wehrli et al., 2001). Importantly, hypoxia-ischaemia is a known risk factor in AD (Zhang and Le, 2010), so a better understanding of clusterin’s function in this context could provide valuable insight into the contribution of clusterin to AD pathology. In light of our preceding discussion it is also clear that we need to know more about which clusterin species are found in the disease models. In this sense, *CLU*-KO is a blunt tool since both secreted and intracellular clusterin is abolished, thereby blinding us to any function(s) performed by specific species. This concept is explored further in our discussion of the role of clusterin in cancer (see section “Clusterin and Cancer”).

### Clusterin and Cancer

Much of what we currently understand about clusterin arises from oncology research. Clusterin is overexpressed in a variety of cancers including breast (Ranney et al., 2007; Yom et al., 2009), ovarian (Xie D. et al., 2005; Wei et al., 2009), and prostate cancer (July et al., 2002), where it promotes tumorigenesis and contributes to chemoresistance (Cao et al., 2005; Flanagan et al., 2010; Kususda et al., 2012; Tang et al., 2012; Xiu et al., 2013). Oncology research has highlighted the opposing roles of clusterin proteins in cell death and survival, presenting the same apparent contradiction as seen in neurodegeneration. Here, we will discuss the current knowledge of clusterin’s role in these pathways, and how research from oncology may lend insight into the role of clusterin in AD.

Initial suggestions gave rise to the idea that sCLU provides a pro-cell survival role while intracellular clusterin proteins are pro-apoptotic (Leskov et al., 2011). This apparent functional dichotomy was thought to arise from the existence of different clusterin proteins with different cellular localizations. An overall increase in *CLU* expression may not be the critical factor in cancer, instead it is thought that an altered ratio of intracellular: secreted CLU proteins may be the key factor, such that cancer is accompanied by an increase in CLU secretion and a reduction in intracellular CLU (Pucci et al., 2004). Most oncology research in clusterin focuses on the role of sCLU, since it is believed to promote chemoresistance (Cao et al., 2005; Flanagan et al., 2010; Kususda et al., 2012; Tang et al., 2012; Xiu et al., 2013) and survival of cancer cells through both anti-apoptotic and pro-survival mechanisms (Ammar and Closset, 2008; Shim et al., 2011; Wang et al., 2013). Numerous studies have shown that the knockdown of *CLU* increases cell sensitivity to chemotherapy drugs and enhances cell death (Viard et al., 1999; Lee et al., 2002; Trougakos et al., 2004; So et al., 2005; Redondo et al., 2007). Although these studies have not specifically knocked down expression of either secreted or intracellular clusterin, it is likely, given the postulated origins of intracellular clusterin, that knockdown of *CLU* will result in a reduction of secreted clusterin and thus, also intracellular clusterin. The potential therapeutic advantage of silencing *CLU* in cancer has been examined in clinical trials. Custirsen (OGX-011) is an antisense oligonucleotide that targets CLU (Chi et al., 2005; Zielinksi and Chi, 2012). The antisense design strategy was aimed at specifically targeting secreted clusterin and had no reported effect on nuclear clusterin expression (Cao et al., 2005), but nevertheless, from the oligonucleotide design it would not be able to distinguish between sCLU and intracellular forms of CLU unless altered translation initiation start sites are used. However, addition of Custirsen to the current treatment regime for prostate cancer does promote sensitization of cancer cells during chemo-therapy (He et al., 2009; Saad et al., 2011; Kususda et al., 2012; Laskin et al., 2012), but effects were no better than the current regime alone (Beer et al., 2017; Chi et al., 2017).

### Clusterin and Cardiovascular Disease

As discussed above, clusterin possesses both pro-survival and pro-apoptotic functions and is cytoprotective in tumor cells. Clusterin’s role in the heart appears more straightforward and is considered primarily protective in cardiac cells during damage and disease. *CLU* expression is frequently observed to rise in the heart after damage (Swertfeger et al., 1996; Ishikawa et al., 1998; McLaughlin et al., 2000; Miyata et al., 2015; Liu et al., 2018). This rise in clusterin is a potential prognosis marker for heart damage: post-heart transplant patients that recover show increased plasma clusterin compared to those who do not recover (Hollander et al., 2014). Strong evidence suggests that clusterin’s protective role in the heart is attributed to its function as a lipid and fat transporter. Firstly, clusterin is only expressed in damaged arteries in the early stages of atherosclerosis, where it may be acting to increase fat and lipid transport, and not in healthy arteries (Ishikawa et al., 1998); secondly, clusterin and APOE form HDL particles in blood plasma together with APOA-I and paraoxonase (Baralla et al., 2015), promoting their transport and processing in the liver (Rizzi et al., 2009); thirdly, clusterin removes cholesterol from macrophage-foam cells, which are a key cell type in atherosclerotic lesions (Gelissen et al., 1998). This is not to say that the anti-apoptotic and anti-oxidant functions of clusterin do not also contribute to its protective role. Clusterin has been shown to inhibit apoptosis and to protect cardiac cells from ischaemic-reperfusion after heart transplantation (Liu et al., 2018), and it has been associated with a reduction in pro-inflammatory signals such as TNF-α and pro-apoptotic BAX, and with an increase in anti-apoptotic gene BCL-XL (Jun et al., 2011; Foglio et al., 2015; Liu et al., 2018). Clusterin may also act as an anti-oxidant in the heart (Mackness et al., 1997), a function that appears dependent on the phosphorylation of Akt/GSK-β and PI3K pathways (Jun et al., 2011; Liu et al., 2015).

So, do these observations of diverse disease states provide any gestalt perspective of clusterin function? Clearly clusterin is involved in regulating cell death in neurological disease, cardiovascular disease and cancer but whether clusterin is protective or pro-apoptotic requires a more nuanced answer. It is clear from both oncology and cardiovascular research that clusterin has a protective function. In the heart, clusterin’s protective function is attributed to its ability to bind lipids and fats, combined with its anti-apoptotic and anti-oxidant properties. However, these same ‘protective’ functions give clusterin a pathological role in oncology, contributing to both tumorigenesis and treatment resistance. The older view that a duality of function may reflect the actions of different extracellular and intracellular clusterin species, each with distinct structure, function, and location has given way to the realization that sCLU is likely the source of intracellular clusterin. Whether this is due to stress-induced translocation of secreted clusterin, or immature/improperly modified versions of secreted clusterin diverting the protein from the secretory pathway resulting in its accumulation within the cell (and a subsequent loss of secreted clusterin being released from the cell) or whether extracellular sCLU becomes internalized is still unclear. So, for instance, in cancer it may not be the overall rise in clusterin levels that determines its role but the altered location of clusterin proteins resulting in an increased ratio of sCLU to intracellular CLU (Pucci et al., 2004). Interestingly, something similar has been observed in AD, whereby clusterin trafficking has been altered by both Aβ treatment (Killick et al., 2014) and *CLU* AD-mutations (Bettens et al., 2015), resulting in increased intracellular clusterin and a reduction in secreted clusterin. These observations suggest that an alteration in the distribution of clusterin inside and outside of cells may contribute to AD pathogenesis. Additionally, the demonstration that *CLU* silencing provides protection from Aβ-induced neurotoxicity in rodent neurons and iPSC-neurons (Killick et al., 2014; Robbins et al., 2018) also points to the importance of altered distribution of clusterin across the cell and clusterin’s pathological role in mediating Aβ toxicity in neurons. This brings into sharp focus the relevance of understanding how intracellular clusterin arises in cells, the structural differences and similarities between secreted and intracellular clusterin, and how these contribute to pathophysiological events. Resolving these issues is critical not only to understanding the role of clusterin in AD but also to exploiting the therapeutic potential unveiled by *CLU* knockdown while preventing unwanted off-target effects.

## Clusterin and Disease Mechanisms

Given the wide distribution of clusterin in the body and its varied functions, it is not surprising that clusterin is implicated in several diseases. Even though clusterin might affect specific pathways in each of these diseases, its dysregulation appears to be driven by common underlying processes, prominently cell death and survival, and oxidative stress.

### Cell Death and Survival

**FIGURE 2:**
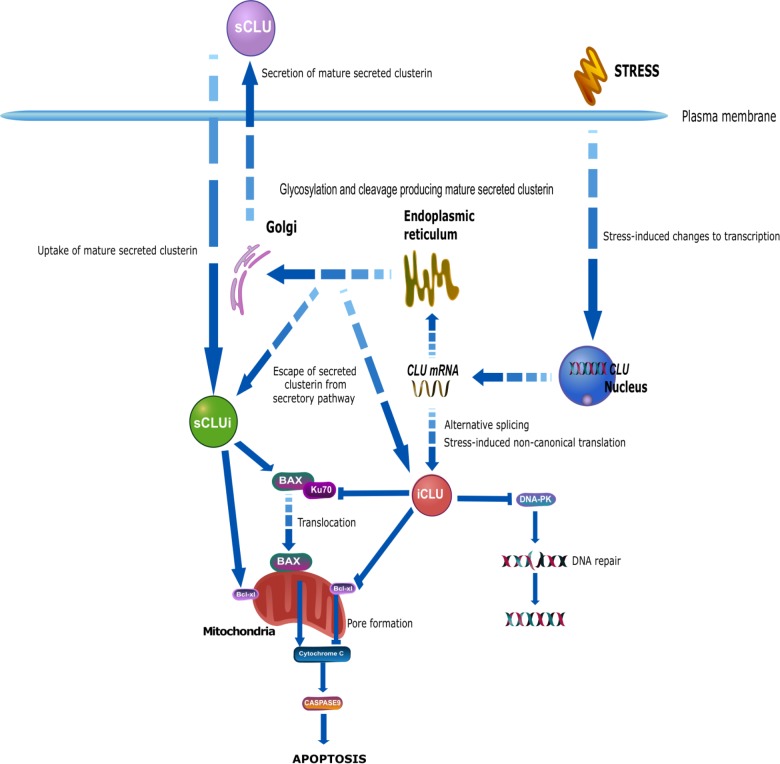
The biogenesis of mature, secreted clusterin involves the traditional secretory pathway common to other secreted proteins. *CLU* is transcribed into the mRNA NM_001831.3, containing exons 1–9. Within exon 2 is a 22-amino acid signal peptide that targets the preproprotein (NP_001822.3) to the endoplasmic reticulum (ER) and the Golgi apparatus. This immature preproprotein undergoes a series of modifications including phosphorylation, cleavage, and extensive glycosylation. Finally, mature secreted clusterin (sCLU) is produced and secreted from cells. Although traditionally referred to as a secreted protein, a number of reports have shown the existence of clusterin inside the cell, referred to as intracellular clusterin. In comparison to secreted clusterin, which has a well described biogenesis, the origin and production of intracellular clusterin is much less clear. Although originally believed that intracellular clusterin arose from a distinct *CLU* mRNA transcript to sCLU, it is now extensively believed that these two proteins share the common mRNA transcript NM_001831.3 and that intracellular clusterin may exist due to altered localization of secreted clusterin. Stress may impair a cell’s ability to secrete sCLU, resulting in its localization within the cell. Alternatively, sCLU may not undergo canonical biogenesis, resulting in incomplete glycosylation and/or cleavage, and this may lead immature sCLU to escape the secretory pathway and remain inside the cell. Additionally, secreted clusterin may be taken into cells by uptake mechanisms resulting in the presence of mature, glycosylated sCLU within the cell. Alternative theories suggest that *CLU* mRNA may undergo stress-induced alternative splicing and non-canonical translation to produce a truncated, non-glycosylated clusterin protein that lacks exon 2 and therefore does not become secreted, but instead accumulates within the cell. Research from oncology indicates a pro-survival function of secreted clusterin and a pro-apoptotic function of intracellular clusterin. Secreted clusterin interacts with the BAX-Ku70 complex, stabilizing it thereby inhibiting the translocation of BAX to the mitochondria where it would promote apoptotic pathways. In contrast, intracellular clusterin competes with BAX for binding with Ku70 and thereby inhibiting their complex formation, resulting in increased free BAX that can translocate to the mitochondria. Secreted clusterin is also thought to interact with Bcl-xl proteins promoting its anti-apoptotic function, whereas intracellular clusterin interacts with this same protein to reduce its anti-apoptotic function. Intracellular clusterin has also been shown to interact with DNA-PK complexes and thereby inhibit DNA repair, resulting in DNA damage and cell stress and death. This figure was produced using icons in the icon library of the Reactome database that are free for download and modification: (https://reactome.org/icon-lib) Accessed August 28, 2018; (Sidiropoulos et al., 2017).

Both secreted and intracellular clusterin have been reported to interact with common proteins in survival and apoptosis pathways, including BAX and Ku70, mediators of BAX-dependent apoptosis (Figure 2). BAX is a key regulator of apoptosis and translocates from the cytosol to the mitochondria during cellular stress whereupon it stimulates pore formation in the mitochondria membrane, enabling the release of pro-apoptotic proteins, thereby promoting cell death (Green and Kroemer, 2004; Moldoveanu et al., 2014). In healthy cells, cytosolic Ku70, a DNA repair protein, binds to BAX to form a complex that inhibits BAX movement to the mitochondria. sCLU can alter this interaction by binding to the complex of Ku70-BAX to stabilize it, thereby reducing the amount of free BAX that can translocate to the mitochondria to induce apoptosis (Zhang et al., 2005; Trougakos et al., 2009). Depletion of clusterin activity has been shown to reduce the binding of Ku70-BAX and increase BAX mitochondrial levels (Trougakos et al., 2009) with the resulting accumulation of pro-apoptotic proteins, increased release of cytochrome C, and increased caspase 9 expression. This is accompanied by a reduction in anti-apoptotic proteins, including Bcl-2, resulting in cells becoming more vulnerable to the pro-apoptotic effects of BAX (Trougakos et al., 2009). Since sCLU is usually located in the extracellular space, the mechanism enabling this intracellular interaction remains unclear. However, internalization of clusterin may occur after binding to receptors such as the megalin receptor (Ghiso et al., 1993; Zlokovic et al., 1996), and several groups have reported glycosylated clusterin protein in the cytosol during cell stress (Nizard et al., 2007), which supports the feasibility of this intracellular interaction between sCLUi, Ku70, and BAX to reduce cell death (Zhang et al., 2005). In contrast, there is some indication that intracellular clusterin may promote apoptosis. Previously Leskov et al. (2003) described nuclear clusterin as a cell death signal and it is thought that nuclear clusterin binds directly to Ku70 (Yang et al., 2000; Leskov et al., 2003), blocking the interaction between Ku70 and BAX, therefore promoting BAX translocation to the mitochondria where it can promote cell death. The interaction and binding with Ku70 were observed with a 60 kDa clusterin protein but not with mature, secreted clusterin and therefore was suggested to be specific to intracellular clusterin (Yang et al., 1999, 2000). The structure or identity of this 60 kDa clusterin protein was not determined and may in fact represent immature secreted clusterin or a modified version of the mature secreted protein. The origin of these differences in these protein interactions of the clusterin proteins has not been explored but may be in part explained by structural differences, such as the level of glycosylation of clusterin, which is known to influence chaperone activity (Rohne et al., 2014). Structural differences between these proteins may therefore be key in explaining the dysfunctionality of clusterin in apoptosis and cell death pathways, as opposed to the existence of different clusterin proteins with separate cellular localizations.

Other pro-apoptotic roles of clusterin in promoting cell death are mediated via interaction with Bcl-xl, an anti-apoptotic protein located on the mitochondrial membrane which is believed to contribute to the regulation of the permeability of the outer mitochondrial membrane (Kelekar and Thompson, 1998; Youle and Strasser, 2008) and the release of pro-apoptotic proteins including cytochrome C, resulting in cell death (Kim et al., 2012). In the heart, cardiomyocytes are protected from H_2_O_2_-induced apoptosis if they are treated with clusterin; cells show increased expression of Bcl-2 proteins and reduced BAX expression, and clusterin-treated cells also show increased protection from H_2_O_2_-induced cell death (Jun et al., 2011). After heart transplantation, clusterin expression reduces the expression of inflammatory proteins including TNF-α and BAX, additionally increasing Bcl-xl expression both *in vivo* and *in vitro*. This reduces cell death and ischaemia reperfusion injury after heart transplantation, a key source of heart damage (Liu et al., 2018). Overexpression of intracellular clusterin cDNA fragments reduces cell viability, which can be rescued by overexpression of Bcl-xl and inhibition of caspases (Debure et al., 2003). nCLU has also been implicated in DNA repair pathways. In fact, nCLU was the first stress-inducible protein shown to be associated with the DNA-dependent protein kinase complex (DNA-PK) during double-stranded break repair of DNA, an association that prevents DNA repair resulting in cell death (Leskov et al., 2001). Currently the contribution of nCLU to cell death and DNA repair mechanisms is not fully understood. Additionally, cytosolic clusterin inhibits NF-κB-dependent Bcl-xl expression and induces arrest of the cell cycle promoting cell death (Scaltriti et al., 2004a,b; Zhang et al., 2005; Takase et al., 2008; Kim et al., 2012). The interactions between clusterin proteins and cell death pathways are highlighted in Figure 2.

Not all clusterin proteins have been observed to influence BAX-mediated apoptosis. Prochnow et al. (2013) observed that non-secreted clusterin proteins observed after proteasomal inhibition did not influence BAX apoptosis. In addition to interactions with apoptotic pathways, there is some evidence supporting interaction of clusterin with pro-survival pathways. PI3K activates and phosphorylates Akt where it localizes to the plasma membrane and can influence several downstream effectors such as mTOR. In several cell lines, clusterin has been observed to interact with this pathway and promote cell survival. In the heart, clusterin protects H9c2 cardiomyocytes from oxidative stress-induced cell death, possibly by regulation of PI3K-Akt and GSK-3β signaling (Jun et al., 2011). This pathway is also active in retinal cells (ARPE-19) where cell death induced by H_2_O_2_ was reduced by clusterin, an effect that that was blocked by inhibition of the PI3K-Akt pathway (Kim et al., 2010). This interaction is also observed in the induced prostate cancer cell line, MLLTet-sClu, where the same dose of doxycycline that was enough to induce clusterin synthesis, increases Akt and Bad phosphorylation, and reduces cytochrome C release, thus promoting cell survival (Ammar and Closset, 2008). Although not much is known about the role of clusterin in mediating cell survival pathways directly, the combinatory role of clusterin in both an anti-apoptotic role and in the enhancement of pro-survival pathways suggests a key role for clusterin in promoting cell survival during cellular stress.

### Oxidative Stress

Oxidative stress is the result of an imbalance between the production of reactive oxygen species (ROS) and the body’s defenses to remove them, and is implicated in many disorders including cancer (Campisi and Fleshner, 2003), cardiovascular diseases (Stocker, 2004), and numerous neurodegenerative diseases including Parkinson’s disease (Lin and Beal, 2006) and AD (Praticò et al., 2002; Honda et al., 2004; Mattson, 2004). In the aging brain, oxidative stress is increased (Finkel and Holbrook, 2000; Golden et al., 2002) and the AD brain is characterized by increased oxidative stress markers including DNA damage and lipid peroxidation (Praticò et al., 2002), which can result in cell death (Butterfield et al., 2002). ROS are produced naturally in the body by the interaction of oxygen with redox metal ions such as copper and zinc. Aβ produces a large amount of ROS through activation of NMDA receptors (Parks et al., 2001; De Felice et al., 2007; Shelat et al., 2008), which may contribute to synaptic dysfunction (Hu et al., 2009; Rönicke et al., 2011). Interaction between Aβ and redox metal ions is also observed; Cu^2+^ and Aβ produce H_2_O_2_ (Huang et al., 1999; Cuajungco et al., 2000; Opazo et al., 2002) and Aβ toxicity is enhanced in the presence of Cu^2+^ (Opazo et al., 2002). Clusterin is thought to be a sensitive biosensor of oxidative stress (Trougakos and Gonos, 2006) since a number of sites in the *CLU* promoter are responsive to changes in oxidative stress (Herault et al., 1992; Wong et al., 1993; Michel et al., 1995; Jin and Howe, 1997), including proteolytic stress-mediated increases in *CLU* expression via binding of both HSF-1 and HSF-2 to a CLE in the *CLU* promoter (Chondrogianni et al., 2003; Loison et al., 2006). Oxidants such as H_2_O_2_ can alter *CLU* mRNA expression and sCLU protein levels (Frippiat et al., 2002) whilst ionizing radiation-induced oxidative stress leads to the accumulation of sCLU at low doses and nCLU at higher, toxic doses (Leskov et al., 2003; Criswell et al., 2005). Numerous studies in various cell types suggest a protective role of sCLU against oxidative stress. For instance, in the heart, sCLU accumulates in arterial walls during the early stages of atherosclerosis and is thought to be a protective mechanism against oxidative stress (Mackness et al., 1997). sCLU also protects cancer cells from H_2_O_2_ and UVA radiation (Viard et al., 1999; Dumont et al., 2002; Miyake et al., 2005). Time-dependent increases in *CLU* mRNA and protein are also observed in neuroblastoma cells under conditions of lipid peroxidation and production of ROS, suggested to be a protective mechanism inhibiting cellular damage during oxidative stress (Strocchi et al., 2006). The silencing of *CLU* in human cancer cells increases apoptosis, reduces growth and increases vulnerability to oxidative stress (Trougakos et al., 2004).

Overall, clusterin appears to be protective against oxidative stress and provides both short term resistance to oxidative stress-induced damage and also long-term survival. In common with the manifold functions of clusterin the exact contributions of sCLU and intracellular clusterin to protection against oxidative stress remain unclear. Oxidative stress is a critical factor observed both during normal aging and in neurodegenerative diseases like AD. A loss of sCLU’s protective role against damage induced by ROS may underwrite increased vulnerability of neurons to insults such as Aβ, which produces both H_2_O_2_ and other ROS. If sCLU is the critical player then the observed reduction in sCLU after Aβ treatment in neurons may result in a loss of protection against oxidative stress and cell death.

## Clusterin (dys)Function in AD

### Clusterin and Aβ Metabolism

The relationship between clusterin and Aβ has been reported for over two decades (Ghiso et al., 1993), with pioneering work already showing that clusterin prevented Aβ aggregation in an *in vitro* acellular system (Matsubara et al., 1996). Around that time, LRP2 (also known as megalin/gp330) was identified as an endocytic receptor for clusterin (Kounnas et al., 1995) and was shown to mediate CLU-Aβ transport through the blood–brain barrier (BBB) and through the blood-CSF barrier (Zlokovic et al., 1996). Despite these initial observations, the anti-amyloidogenic nature of clusterin continues to be a matter of controversy fuelled by the confounds arising from use of numerous and diverse biological and disease models.

#### Effects on Amyloid Toxicity

A common way of studying the effect of clusterin on Aβ aggregation and resulting toxicity has been to incubate amyloid preparations with or without clusterin in different biological systems. The use of different Aβ species, aggregation protocols and incubation strategies has undoubtedly led to the variable downstream biological effects that have been observed. Nevertheless, a common finding is that clusterin interferes with Aβ aggregation, in agreement with observations from Narayan et al. (2012) showing that clusterin is able to bind a wide range of Aβ oligomers (from dimers to 50-mers), and consequently to prevent further aggregation into fibrils. However, this interference with Aβ aggregation has been observed to influence toxicity of the amyloid products in differing ways. On the one hand, clusterin’s cytoprotective effects were detected in a study with SH-SY5Y cells treated with Aβ-supplemented AD CSF, where the addition of a mix of extracellular chaperones including clusterin into the CSF preserved cell viability (Yerbury and Wilson, 2010). Similarly, co-culture experiments on rat hippocampal astrocytes and neurons showed that clusterin incubation prevents Aβ-induced astrocytic calcium uptake, resulting in decreased ROS generation and caspase 3 activation; additionally, clusterin blocked Aβ-induced inhibition of LTP on hippocampal slices (Narayan et al., 2014). In another study, incubation of Aβ aggregates with clusterin before injection into rat brains improved performance in the Morris water maze test and decreased glial activation and neuronal degeneration compared to that observed in rats injected with Aβ42 alone or clusterin and Aβ42 with no previous incubation (Cascella et al., 2013). On the other hand, reports show an increase in the amount of more diffusible, soluble oligomeric species and reduced fibril formation resulting in increased cellular oxidative stress (Oda et al., 1995) and neurotoxicity in organotypic mice brain slices (Lambert et al., 1998). In a conciliatory study, the duality of clusterin’s effect on Aβ toxicity was postulated to be dependent on the molar ratio of clusterin and Aβ (Yerbury et al., 2007). Clusterin reduced Aβ aggregation and toxicity but, when Aβ was present in an excess compared to clusterin, there was an increase in amyloid formation. In the latter, clusterin was also incorporated into these amyloid aggregates, which were more toxic than aggregates containing Aβ42 alone. Given that concentrations, timescale, and tissue location of the clusterin-Aβ interaction may vary significantly between the AD brain and these simplified experimental models, the relevance of these observations to AD pathology remains to be proven.

#### Effects on Amyloid Clearance

Several mechanisms of brain Aβ clearance have been described, including intracellular uptake and transport across the BBB (Tarasoff-Conway et al., 2015), both of which have implications for the role of clusterin in AD.

Astrocytes and microglia are both implicated in capturing and eliminating Aβ from the brain interstitial fluid (ISF) (Tarasoff-Conway et al., 2015). Again, the role of clusterin on these interactions is far from clear. Treatment of human astrocytes with Aβ results in a simultaneous increase of intracellular clusterin, decrease of secreted clusterin, and appearance of vacuoles containing fibrillary Aβ, arguing in favor of clusterin’s role in promoting Aβ clearance (Nuutinen et al., 2007). However, in other studies, incubation of pre-aggregated Aβ with clusterin resulted in reduced amyloid uptake from oligomer-enriched preparations in cultures of human primary astrocytes (Nielsen et al., 2010) and from fibril-enriched preparations by microglia (Mulder et al., 2014). Interestingly, these studies showed that, despite both cell types being more efficient at taking up amyloid from oligomer-enriched rather than from fibril-enriched preparations, microglia had a higher capacity for binding and internalizing amyloid from a fibril-enriched preparation than astrocytes, which suggests different roles and mechanisms of Aβ clearance by microglia and astrocytes (Mulder et al., 2014). Another study observed that incubation of AD CSF with clusterin actually increased the removal of Aβ42 from the supernatant by macrophage-like U937 cells (Yerbury and Wilson, 2010). More recently, it was described that mouse primary microglia and human monocyte-derived macrophages use TREM2 as a receptor to bind and take up lipoproteins and apolipoproteins including clusterin, and that internalization of Aβ is more efficient when complexed with LDL or clusterin (Yeh et al., 2016). *TREM2*, like *CLU*, is an AD-risk locus (Guerreiro et al., 2012; Jonsson et al., 2012). All of this underlies the potential importance of functional links between multiple risk genes in disease mechanism (see section “Relationships to Other AD Genes”).

Clusterin also regulates Aβ transport through the BBB, and Aβ clearance is significantly increased when complexed with clusterin *in vivo*, an effect mediated by LRP2 (Bell et al., 2007). More recent studies have used *in vitro* systems to model a simplified version of the BBB by culturing endothelial cells on a permeable support that allows for separation between apical and basolateral compartments simulating blood and brain, respectively. In a study with mouse primary cerebral endothelial cells, trafficking of fluorescently labeled Aβ40 from basolateral to apical compartment was enhanced when complexed with clusterin, and blockage of LRP1/LRP2 reduced crossing of clusterin between compartments (Merino-Zamorano et al., 2016). In a similar system using porcine brain capillary endothelial cells, exogenous addition of clusterin to the culture increased intracellular protein levels of APP and trafficking of radiolabeled Aβ40 (Zandl-Lang et al., 2017).

These studies indicate that interaction between clusterin and Aβ is potentially important for amyloid clearance. However, these findings also underscore the importance of context when studying this interaction. Different cell-types, varying levels of model complexity, and conditions that represent distinct physiological situations often lead to differing conclusions. Sifting through these confounds to identify the factors that contribute to AD pathology remains a challenge.

#### Effects on AD-Pathology in AD Animal Models

The use of *CLU-*knockout (KO) animal models of amyloidosis has enabled the study of the relationship between clusterin and Aβ *in vivo*. These studies, unsurprisingly, have not provided clear answers on the anti-amyloidogenic nature of clusterin. In seminal work by DeMattos et al. (2002), plaque formation was reduced in *CLU*-KO PDAPP mice compared to *CLU*-expressing PDAPP mice. Plaques formed in the absence of *CLU* displayed decreased surrounding neuritic dystrophy, which argued in favor of a pro-amyloidogenic role of clusterin in this mouse model (DeMattos et al., 2002). However, subsequent work by the same group showed that knocking out both *CLU* and *APOE* in PDAPP mice resulted in increased Aβ production and amyloid deposition and earlier onset of AD-pathology than the *APOE*-KO alone (DeMattos et al., 2004). These findings suggest that clusterin and APOE may work cooperatively in this mouse model to reduce amyloid deposition and illustrate the complexity of understanding Aβ-clusterin interactions *in vivo.* A more recent study in APP/PS1 mice showed that *CLU*-KO shifts deposition of Aβ from plaques to accumulation in the cerebrovasculature, resulting in increased amyloid angiopathy but, surprisingly, reduced hemorrhage and inflammation (Wojtas et al., 2017). These varied findings across *CLU*-KO mice with different genetic backgrounds illustrate the need to consider potential compensatory mechanisms and parallel pathways when drawing conclusions from a KO model, and offer a cautionary note on the even greater distance that might exist in clusterin function between animal models and humans.

### Clusterin in DKK1-Driven Altered Wnt Signaling

In addition to its direct interactions with Aβ and the resulting downstream effects, clusterin has been implicated in Wnt signaling, a pathway that has been intensely scrutinized in relation to AD. Aβ treatment of neurons gives rise to a neurotoxic response and an associated upregulation of dickkopf 1 (DKK1), an antagonist of canonical Wnt signaling (Killick et al., 2014), which leads to an upregulation of GSK-3β, increased tau phosphorylation (Caricasole et al., 2004) and synapse loss (Purro et al., 2012). *CLU* knockdown in cultures of rat primary cortical neurons prevents the Aβ-driven increase in *DKK1* expression levels and protects cells from Aβ-induced neurotoxicity (Killick et al., 2014). Furthermore, treatment of cells with Aβ increases intracellular levels of clusterin while decreasing release of extracellular, secreted clusterin, similarly to what has also been described in astrocytes (Nuutinen et al., 2007). These changes took place within 30 min and in the absence of any change in *CLU* mRNA expression (Killick et al., 2014), suggesting that changes in clusterin are post-transcriptional and primarily result from changes in clusterin secretion. Further characterization of this pathway led to the postulation of a switch in Wnt signaling to the non-canonical Wnt-PCP-JNK pathway, and to the subsequent activation of downstream transcription factors upregulated by both Aβ and DKK1 – an upregulation prevented by *CLU*-knockdown. When individually silenced, these genes protected cultures from Aβ-induced neuronal cell death (*EGR1* and *KLF10*) and/or restored phosphorylated tau (p-tau) down to basal unstimulated levels (*EGR1* and *NAB2*) (Killick et al., 2014). Moreover, a very recent study from our lab performed in human induced pluripotent stem cell (iPSC)-derived neurons gives further support to clusterin mediating Aβ toxicity (Robbins et al., 2018), showing neurite length after Aβ-insult is preserved in *CLU*-KO cells. Interestingly, previous reports had demonstrated co-localization of clusterin and Aβ plaques surrounded by p-tau-positive dystrophic neurites and with p-tau deposits within the neurites in the temporal cortex of AD patients (Martin-Rehrmann et al., 2005). Furthermore, in the AD entorhinal cortex, clusterin co-localized with neurofibrillary tangles (NFTs), and neurons containing NFTs showed increased expression of *CLU* (Dunckley et al., 2006). Both mouse primary cortical neurons treated with clusterin and rats intracerebrally injected with clusterin showed an increase in tau and p-tau levels (Martin-Rehrmann et al., 2005). These studies are in disagreement with initial observations describing an increased proportion of NFT-free neurons expressing *CLU* in AD entorhinal, temporal, and frontal cortices, and clusterin localization in NFT-containing neurons was only rarely observed (Giannakopoulos et al., 1998). Further exploration of the effectors responsible for this neuroprotection is urgently needed, as these hold promise for developing new AD therapeutics.

### Clusterin in Immunomodulation

Changes in the immunological mechanisms in the brain are considered a key component of AD pathogenesis, whereby proteotoxic aggregates trigger an innate immune response that exacerbates disease progression (Heneka et al., 2015). Moreover, recent GWAS have identified several immune genes as risk factors for AD, with a particular enrichment in complement components, highlighting even further the relevance of the innate immune system in AD pathogenesis (Lambert et al., 2009, 2013; Seshadri et al., 2010; Hollingworth et al., 2011; Guerreiro et al., 2013; Sims et al., 2017). The complement system (CS) is part of the innate immune system and recognizes a variety of potentially harmful elements ranging from invading microorganisms to toxic protein aggregates such as amyloid deposits. Through an activation cascade that includes over 30 factors operating in a network of three integrated pathways (classical, alternative, and lectin), the CS is involved in neuro-inflammatory signaling and culminates in assembly of the membrane attack complex (MAC), a multi-component structure that generates pores in the membrane of the targeted cells leading to lysis (reviewed in Veerhuis et al., 2011). The CS is overactive in AD brain, where components of the MAC and several complement factors (C1q, C4, C3) are found not only in association with amyloid aggregates but also with tau inclusions and damaged neurons (McGeer et al., 1989; Shen et al., 2001, 2013). Within its immunomodulatory functions, clusterin is best known for regulating the CS. Clusterin was found to regulate the formation of the MAC, specifically as an inhibitor of C5b-6, the first step in MAC formation (Jenne and Tschopp, 1989; Murphy et al., 1989). Soon after, immunohistochemistry data showed similarities in the staining pattern of clusterin and MAC in brain tissue, both of which localized in dystrophic neurites and neuropil threads in AD but not in control brains, suggesting a potential increase in clusterin levels as a protective response to MAC formation (McGeer et al., 1992). Later on, it was argued that CS-inhibition is not possible at the physiological concentrations at which clusterin is found (Hochgrebe et al., 1999), but additional studies have shown more examples of local clusterin up-regulation leading to CS-inhibition (Urbich et al., 2000; Hallström et al., 2015). Nevertheless, a mechanistic relationship of CS-inhibition by clusterin in the context of AD remains to be investigated. Links between clusterin and other inflammatory processes have been studied, including modulation of NF-κβ signaling (Santilli et al., 2003) and multiple cytokines including TGF-β (Morgan et al., 1995), TNF-α (Shim et al., 2012), and IL-2 (Sonn et al., 2010). In a study with rat microglia, clusterin treatment induced cell morphological activation both *in vitro* and *in vivo* (Xie Z. et al., 2005); additionally, clusterin-activated microglia secreted more reactive nitrogen intermediates and TNF-α, and boosted neurotoxicity when co-cultured with rat primary cortical neurons. The inflammatory component of AD is an area under intense study, and it is clear that glial responses to protein aggregation contribute to AD pathogenesis (Heneka et al., 2015); however, the potential neuro-inflammation regulatory role of clusterin in AD has not been studied in depth. Given the multiple links that have been established between clusterin and several immunomodulatory actors, clusterin could offer some therapeutic potential as a mediator of the boosted immune response observed in the AD brain.

### Clusterin and Copper Homeostasis

Dysregulation of copper homeostasis is a known pathophysiological event occurring in AD (reviewed in Greenough et al., 2016). Copper has a role in limiting the amyloidogenic processing of APP (Borchardt et al., 1999), and accumulation of copper in amyloid deposits, as well as brain copper deficiencies, have been described in AD (Deibel et al., 1996; Lovell et al., 1998). Copper-transporting P-type ATPases (copper-ATPases) ATP7A and ATP7B regulate brain copper transport so that levels are sufficient for copper-associated proteins but also ensuring that toxic copper excess is removed (Telianidis et al., 2013). Experiments performed in the context of Menkes and Wilson diseases, in which mutations in *ATP7A* and *ATP7B* lead to copper deficiency and copper toxicity disorders, respectively, showed that clusterin participates in the degradation of ATP7A and ATP7B (Materia et al., 2011) via the lysosomal pathway (Materia et al., 2012), an observation that tallies with the reported function of intracellular clusterin in facilitating autophagy (Zhang F. et al., 2014). Interaction between clusterin and the copper-ATPases increases under conditions of oxidative stress and by mutations in *ATP7B*, suggesting that oxidative stress caused by a dysregulation of copper levels might be driving clusterin-associated degradation of ATP7A and ATP7B (Materia et al., 2011). Interestingly, two SNPs in high linkage disequilibrium within *ATP7B* (rs1061472 and rs732774) have been linked with increased risk of AD (Bucossi et al., 2012), being the haplotype located in the *ATP7B* regions encoding for functionally important transmembrane and transduction domains (Squitti et al., 2013). Clearly further investigation is needed to fully unravel the potential role of CLU in copper dyshomeostasis observed in AD, but nevertheless these studies do point to an alternative pathway that could be used as a novel therapeutic target.

## *CLU* Genetics and Alzheimer’s Disease

Mutations in several genes have been identified to cause familial AD, but these are rare. Most cases of AD are sporadic and arise due to complex interactions between the environment and risk genes. Heritability of LOAD is estimated to be between 60 and 80%, and the exact contribution that genetics play in LOAD is still unclear (Gatz et al., 2006). Extensive genetic studies have identified genes that alter AD risk and numerous SNPs have also been identified associated with these genes (Bertram et al., 2007; Bertram and Tanzi, 2009; Harold et al., 2009; Lambert et al., 2009, 2013; Seshadri et al., 2010).

### GWAS and CLU

**FIGURE 3:**
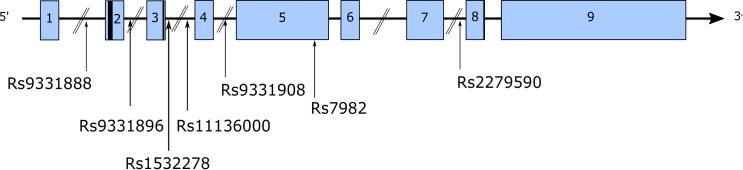
A multitude of SNPs have been identified in *CLU*, both intronic and exonic. The figure highlights a number of these SNPs identified in AD GWAS.

Repeatedly the *APOE* genotype has been identified as the greatest common genetic risk factor for LOAD and until 2009 was the only one collectively recognized by the AD community (Corder et al., 1993; Saunders et al., 1993). However, *APOE* genotype only accounts for an estimated 27% of LOAD heritability (Gatz et al., 2006; Lambert et al., 2013) suggesting other genes must also contribute to LOAD risk. GWAS have now identified several genes that alter LOAD risk including, among others, bridging integrator 1 (*BIN1)* and *CLU*, which are the second and third most common genetic risk factors after *APOE* ^1^. Two large GWAS independently identified a number of SNPs located at the *CLU* locus associated with altered AD risk (Figure 3). Lambert et al. (2009) identified 36 SNPs in *CLU* in control individuals, and 3 (rs11136000, rs93318888, and rs2279590) were associated with altered AD risk. Additionally, these SNPs were observed in a linkage disequilibrium (LD) block, which only encompassed *CLU* (Lambert et al., 2009). Harold et al. (2009) similarly observed a significant association between *CLU* SNP rs11136000 and AD, and this was further replicated in another GWAS (Seshadri et al., 2010) and in smaller Caucasian and European cohorts (Carrasquillo et al., 2010; Naj et al., 2011; Shuai et al., 2015; Zhang S. et al., 2015, 2016; Zhu et al., 2017). Rs11136000 was considered to be the main *CLU* SNP that altered AD risk (Harold et al., 2009; Lambert et al., 2009). However, replication in Asian cohorts has been less consistent (Yu et al., 2010; Ma et al., 2011; Lin et al., 2012; Ohara et al., 2012; Chung et al., 2013; Miyashita et al., 2013; Liu et al., 2014; Du et al., 2016), suggesting other SNPs contribute to AD risk in Asian cohorts. Similarly, less consistent associations have been observed in Turkish populations (Alaylloglu et al., 2016).

### Rs1136000

Located in intron 3 of *CLU*, rs11136000 was considered the major AD risk-altering SNP in *CLU* (Harold et al., 2009; Lambert et al., 2009). The C allele confers an increased risk of 1.16-fold to AD (Bertram et al., 2007) and is carried by 88% of Caucasians (Lambert et al., 2009). In addition to its association with AD, rs11136000 is associated with both Mild Cognitive Impairment (MCI) (Cai et al., 2016) and the progression from MCI to AD (Carrasquillo et al., 2015). Presymptomatic C carrier individuals display increased cognitive decline (Thambisetty et al., 2013), and both AD and the non-demented elderly possessing the C allele show poorer memory scores (Pedraza et al., 2014). In comparison, the minor T allele is thought to confer protection to AD (Lin et al., 2012) and is associated with improved cognitive function in the elderly (Mengel-From and Christensen, 2011). Memory function appears to be altered by rs11136000 allelic expression, as the C allele is associated with poorer neural efficiency and increased limbic and memory area activation during working memory tasks (Lancaster et al., 2015). Although Erk et al. (2011) observed no influence of rs11136000 genotype on brain activity, they observed decoupling of the dorsolateral prefrontal cortex (DLPFC) and the hippocampus during episodic memory recall and the T allele was shown to be associated with reduced hippocampal activity during working memory tasks in healthy controls (Lancaster et al., 2011), although one study observed an increased rate of cognitive decline in individuals with the T allele of rs11136000 (Sweet et al., 2013). Imaging studies are increasingly used to identify potential correlates between genotype and structural and functional changes in the brain. Green et al. (2014) observed an additive effect of *CLU* genotype and *APOE* genotype on brain activity, observing a reduction in brain activity in the young possessing rs11136000-C and *APOE E4* alleles during tasks requiring executive attention. However, results are not consistent as Biffi et al. (2010) failed to find associations between *CLU* rs11136000 genotype and a number of neural measures, which may be due to a small sample size. Correlations between rs11136000 and brain atrophy have been observed (Thambisetty et al., 2012; Haight et al., 2018). Even in non-demented elderly individuals, the C allele appears to influence brain structure; Qiu et al. (2016) observed altered fractional anisotropy (FA) in the left external capsule that was correlated with cognitive scores. Changes in brain structure are observed in the young too, since a reduction in white matter integrity in several areas including the corpus callosum (Braskie et al., 2011), subtle reductions in gray matter volume of the right hippocampal formation (Lancaster et al., 2015), and a bilateral increase in entorhinal cortex volume in young, healthy individuals possessing the C allele have been observed (DiBattista et al., 2014). These studies suggest brain changes occur early in life and may predispose individuals to AD at a later stage.

A functional variant of *CLU* in AD has not yet been identified (Bettens et al., 2010) and much effort has been placed on examining the role of intronic variants on the regulation of genes related to AD (Hardy and Singleton, 2009; Manolio et al., 2009). Research suggests that *CLU* SNPs may influence *CLU* mRNA (Szymanski et al., 2011) and protein expression (Xing et al., 2012; Cai et al., 2016; Tan et al., 2016). However, results from different studies are inconsistent. MCI patients show increased plasma clusterin in the presence of rs11136000-C allele, which negatively correlates with cognitive function scores (Cai et al., 2016). Schurmann et al. (2011) observed a reduction in clusterin plasma in a dose-dependent manner with the rs11136000 C risk allele; however, this was not observed to be significantly different between healthy individuals and AD patients. Similarly, although Mullan (2013) observed higher plasma clusterin in individuals possessing the rs11136000, TT genotype, this was observed in both AD, MCI patients and healthy controls. Ling et al. (2012) described a preferential effect of the T allele of rs11136000 on a single *CLU* mRNA transcript and commented that the protective T allele of rs11136000 is associated with an increased expression of one *CLU* transcript, which may reflect higher sCLU protein levels throughout life that may provide protection.

### Other GWAS SNPs

Other *CLU* SNPs associated with AD risk include: rs9331888 (Lambert et al., 2009; Yu et al., 2010; Gu et al., 2011), rs2279590 (Lambert et al., 2009; Schjeide et al., 2011; Chen et al., 2012; Miyashita et al., 2013), rs7982 (Harold et al., 2009; Jun G. et al., 2011), rs9331908 (Bettens et al., 2012), and rs1532278 (Bettens et al., 2012).

#### Rs9331888

Rs9331888 was originally identified by Lambert et al. (2009), as an exon 1-located SNP and is associated with reduced baseline left hippocampal volume in a subset of AD patients (Tan et al., 2016). Possession of the G risk allele of rs9331888 reduces blood clusterin mRNA and protein in controls and AD patients, but this reduction is much greater in controls (Xing et al., 2012). Conversely, Szymanski et al. (2011) examined the influence of the risk G allele on the expression of *CLU* mRNA transcripts described at the time (NM_001831, NM_20339, and NM_001171138) and observed an increase in the relative abundance of NM_203339 in the temporal cortex of AD patients, suggesting this SNP is regulating alternative splicing of *CLU*. At the time, this transcript was suggested to be translated to form sCLU, but as discussed previously, NCBI annotations have been updated^2^ (accessed August 28th 2018), and it now appears that rs9331888 is located within an intron of the main NM_001831.3 transcript and not in exon 1 of NM_203339 as initially believed. This questions the validity of the observation of rs9331888 as a functional variant by influencing alternative splicing of *CLU.*

#### Rs1532278

Rs1532278 is located in intron 3, like rs11136000, and sits in a NKX2-5 binding motif and affects the binding of NANOG, TAF1, USF1, MAX, USF2, and GATA2. Rosenthal et al. (2014) used the RegulomeDB database to identify AD GWAS SNPs possessing regulatory functions. Out of the 34 SNPs identified in 18 genes previously linked to AD including *CLU, BIN1, PICALM*, and *CD2AP*, only three SNPs showed evidence of regulatory function, one of which was located in *CLU*, rs1532278. Roussotte et al. (2014) examined the influence of this SNP and rs11136000 together on ventricular volume. After both a 1-year and 2-year follow-up period, homozygous individuals for the risk alleles showed greater ventricular expansion. This was not influenced by *APOE E4* genotype and regardless of dementia status. Both SNPs were also associated with longitudinal changes in ventricular volume/expansion (Roussotte et al., 2014).

#### Rs2279590

Rs2279590, located in intron 7, is positioned in an active regulatory region characterized by the presence of H3K27Ac, increased DNase hypersensitivity, and an eQTL for *CLU* (Padhy et al., 2014). The risk allele of this SNP differentially regulates *CLU* expression (Padhy et al., 2014). Enhancer elements flank this SNP and appear to regulate *CLU* expression, while deletion of these elements reduces *CLU* expression (Padhy et al., 2017). Additionally, the non-risk allele at this SNP site forms a transcription factor binding site for HSF-1, which is not present with the risk G allele (Padhy et al., 2017). The functional relevance of a loss of binding site for HSF-1 in the risk allele must be explored. As we have discussed, *CLU* expression is upregulated by a variety of signals including heat shock-induced stress. If the upregulation of *CLU* is protective, the loss of this binding site for HSF-1 may have a functional effect that may contribute to increased AD risk.

#### Rs9331896

Rs9331896, also an intronic SNP, was identified by Lambert et al. (2013) as the most significantly associated *CLU* SNP with AD risk and is in LD with numerous other *CLU* SNPs including rs11136000, rs9331888 and rs2279750 (Ward and Kellis, 2012; Lambert et al., 2013). Additionally, like other CLU SNPs, rs9331896 is significantly associated with altered CLU expression. Karch and Goate (2015) reported that this SNP is located in an eQTL for *CLU* and is associated with *CLU* expression in the white matter of the hippocampus, temporal cortex, and occipital cortex.

To date, rs11136000 is the most studied SNP found in *CLU* and although it was once considered to be the most influential *CLU* SNP affecting AD risk in Caucasian populations, it is now ranked as the second, after rs9331896. A range of *CLU* SNPs have been identified through GWAS, but little is known about their contribution to AD. Despite this, SNPs including rs2279590, rs9331888 and rs1532278 have been observed to have potential roles in regulating gene expression. Interestingly, most of the significant SNPs appear to exist in a linkage disequilibrium block, including rs11136000, rs9331896, and rs2279590, and may together influence risk that is not achieved when acting alone. The functional influence of *CLU* SNPs is unclear but several SNPs are observed to influence *CLU* mRNA expression and the levels of plasma clusterin. Effects are observed both in AD patients and in the young and healthy, suggesting a mechanism by which vulnerability may arise decades prior to disease onset. Although there is some conflicting evidence, replication of GWAS and patient-control studies provides strong evidence that *CLU* variants influence AD risk, and this is independent of *APOE E4* status.

### Relationships to Other AD Genes

The consensus is that GWAS SNPs and mutations have a significant but modest effect on AD risk individually. Even the *APOE E4* genotype only accounts for a portion of LOAD heritability (Gatz et al., 2006), and complex genetic and environmental interactions are likely to contribute to AD development. As previously discussed, SNPs within *CLU* have been shown to interact with each other to influence hippocampal volume (Roussotte et al., 2014), but relationships among different AD GWAS genes and their SNPs are mostly unexplored. Identification of genotype patterns spanning multiple gene loci may be more useful than focusing on one single gene locus. Previously, studies have looked at *PICALM* and *CLU* and their individual and combined influence on brain atrophy and degeneration (Biffi et al., 2010; Erk et al., 2011; Furney et al., 2011; Melville et al., 2012). Barral et al. (2012) identified genotype patterns in *PICALM* and *CLU* that are associated with altered episodic memory performance. No SNP, when tested individually, was shown to be associated with episodic memory performance but different genotype combinations at *PICALM* (rs3851179), *CLU* (rs11136000) and *APOE* (*E3 vs. E4*) were shown to improve or worsen performance. *PICALM-*GG (risk) and *CLU*-TT (protective) had the strongest effect on memory performance, which was worsened further in the presence of *APOE E4*. *PICALM*-AG and *CLU*-CC showed a trend for improvement in memory which only reached significance when *APOE E3* was present (Barral et al., 2012), highlighting that different genotype combinations at three AD genes produce different effects on a measurable phenotype that is AD-relevant. Interestingly, the genotype that is associated with poor memory includes a risk allele for *PICALM* but a protective allele for *CLU* (Lin et al., 2012). These observations suggest that when isolated, a SNP may be protective, but may show different effects on phenotypes in the presence of other SNPs and contribute to disease vulnerability. Similarly, Yang et al. (2016) observed that when *PICALM* rs3581179 (AA) protective genotype was present alongside risk genotype at *CLU* rs11136000 (CC) there was a detrimental effect to memory performance, which was not present when the *CLU* protective genotype was present (TT). It is therefore simply not enough to describe a SNP as protective or risk, without considering which other SNPs are present at different loci.

Hippocampal connectivity appears altered by both *PICALM* and *CLU* genotypes (Zhang P. et al., 2015). Zhang P. et al. (2015) observed in young, healthy Chinese Han individuals that *PICALM* weakened hippocampal connectivity (measured as resting state functional brain connectivity) while risk variants in *CLU* had a strengthening effect consistent with the reports of others (Lancaster et al., 2011). This may explain in part why *PICALM* risk genotype is associated with earlier cognitive decline (Sweet et al., 2013) and *CLU* risk genotype is associated with increased rate of decline (Sweet et al., 2013; Thambisetty et al., 2013). This interaction between *PICALM* and *CLU* genotypes is specific to hippocampal connectivity, as no influence has been observed on hippocampal volume (Bralten et al., 2011; Zhang P. et al., 2015) or pathological burden (Chibnik et al., 2011).

As well as genetic interactions between *CLU* and other AD-relevant genes, biological interactions have also been observed. Clusterin and APOE are both members of the apolipoprotein family of proteins, binding to lipids and cholesterol to promote their transport and processing, an important part of their homeostasis (Mahley, 1988; De Silva et al., 1990). Thus, it is not surprising that many comparisons have been made between *APOE* and *CLU* (Kounnas et al., 1995; Koch et al., 2001). The exact mechanisms by which these genes influence AD risk are unclear. It is also unclear whether they have a shared mechanism or interact to influence risk. For example, Green et al. (2014) observed that *CLU* variants influence brain activity in an additive manner in the presence of *APOE E4* genotype, resulting in a reduction of temporal lobe brain activity in young, healthy individuals. *APOE* genotype is the key genetic risk factor influencing LOAD risk. There are three identified alleles of *APOE (E2, E3*, and *E4*) and while the *E4* allele increases AD risk, the *E2* allele reduces it. Between the alleles there are two isoform-specific amino acid differences and therefore these variants are described as missense mutations. These differences have direct effects on APOE protein structure and function and can be described as functional variants. In comparison, common variants in *CLU* are intronic SNPs in most cases, and therefore, unlikely to have an influence on protein structure or function. Despite their similarities, it is unlikely that the mechanism by which individual variants influence AD risk will be identical. Association signals observed between *CLU* and AD (Harold et al., 2009; Lambert et al., 2009) are unlikely to be due to common coding variants in *CLU* (Guerreiro et al., 2010) and large expression quantitative trait locus (eQTLs) are not found in *CLU* (Guerreiro et al., 2010; Holton et al., 2013). Some non-synonymous, rare coding mutations have been identified in AD patients, which were found clustered in exons 5–8 of the clusterin β-chain (Bettens et al., 2012). These mutations were observed to influence the secretory trafficking of clusterin and the subsequent secretion from cells in culture (Bettens et al., 2015). One such mutation (P.I03030Nfsx13), a single nucleotide insertion after amino acid residue 315, is predicted to introduce a premature stop codon resulting in total ER retention of clusterin and a loss of sCLU in HEK cells (Bettens et al., 2015). Additionally, rare mutations are observed to increase the generation of intracellular clusterin protein in cells (Zhou et al., 2014). This may have the effect of reducing the protective properties conferred by sCLU and contribute cellular vulnerability to apoptotic stress and protein aggregation.

Recently, both APOE (Atagi et al., 2015) and clusterin have been identified as triggering receptor expressed on myeloid cells (*TREM2)-*binding ligands and facilitate microglial uptake of Aβ (Yeh et al., 2016). TREM2 is a receptor selectively expressed by microglia in the adult CNS (Klesney-Tait et al., 2006; Zhang Y. et al., 2014) and also is an AD risk gene (Guerreiro et al., 2012; Jonsson et al., 2012). The interaction of APOE, clusterin, and TREM2 is impaired by *TREM2* AD mutations and *TREM2* KO (Yeh et al., 2016), including the loss-of-function R47H mutation (Wang et al., 2015), and the lipidation of Aβ with APOE and CLU facilitates uptake by microglia, which is impaired in *TREM2* KO microglia (Yeh et al., 2016). There has been no indication as to whether *CLU* SNPs or mutations influence this relationship. Identification of a variant that would influence this relationship would be a strong functional candidate in *CLU*, since impairments in this would lead to a reduction of Aβ uptake and an increased probability of aggregation. Also recently, plexin A4 (PLXNA4) was identified as a novel receptor for clusterin in the adult CNS, and SNPs observed in this gene were linked to altered CSF clusterin levels (Kang et al., 2015). *PLXNA4* had previously been found as an AD GWAS hit (Jun et al., 2014), and some of its genetic variants have been associated with CSF Aβ42 levels (Han et al., 2018).

After APOE, Bridging Integrator 1 or *BIN1* is the second most common genetic risk factor for LOAD. Alternative splicing produces several isoforms with differing tissue expression and function (Wigge et al., 1997; Pineda-Lucena et al., 2005), and a number of neuronal specific BIN1 isoforms have been identified, which are thought to be involved in endocytosis and clathrin interactions (McMahon et al., 1997; Ramjaun and McPherson, 1998). BIN1 interacts with tau protein and its main influence on AD risk is in the modulation of tau pathology (Chapuis et al., 2013). Overexpression of intracellular clusterin and tau in cell culture results in an interaction between intracellular clusterin and tau, and between intracellular clusterin and BIN1 (Zhou et al., 2014). This interaction was deemed to be specific between intracellular clusterin and neuronal isoforms of BIN1, and is inhibited in the presence of previously identified rare AD mutations (Bettens et al., 2012). A frameshift mutation resulted in a lack of the C terminus coiled-coil motif, which is thought to be essential for the interaction of intracellular clusterin and BIN1 (Zhou et al., 2014). The exact physiological importance of the biological interactions between BIN1 and clusterin is unknown, as is the relevance of the interaction between BIN1, clusterin and tau together – is this interaction affected by more common variants and is it important in disease? It is also unclear how *CLU* or *BIN1* SNPs or variants may influence this interaction, and although not much evidence for *CLU* variants and tau interactions has been observed, the rs11136000 SNP has been correlated with tau expression in the CSF of AD patients (Zhou et al., 2014).

Analyses of GWAS AD genes have highlighted a significant overrepresentation of genes found in common disease pathways such as inflammation and endocytosis (Bertram et al., 2007; Jones et al., 2010), resulting in GWAS AD genes being grouped together based on their similar functions (Rosenthal et al., 2014). For example, both APOE and CLU bind lipids and cholesterol to influence their trafficking, while clusterin and CR1 are regulators of the immune response. Clusterin and PICALM possess differing roles in Aβ metabolism: clusterin promotes clearance (Cirrito et al., 2008) while PICALM promotes deposition (Mengel-From and Christensen, 2011); however, at the genetic level their SNPs appear to interact and influence memory (Barral et al., 2012).

In summary, although its functions are still debatable, it is clear that *CLU* is a key genetic risk factor for LOAD, and a number of intronic and exonic SNPs have been identified, as well as several rare coding mutations. In most cases, little is known about how these SNPs influence mRNA and protein expression of clusterin and no functional variant has yet been identified. However, there is strong evidence to suggest that clusterin protein interacts with a number of other AD-relevant proteins including the main AD pathological proteins such as tau and Aβ, as well as AD risk factors such as *APOE* and *BIN1*. Although these interactions have been observed at a biological level, the influence of genetic variants in *CLU* on these interactions remains elusive. Investigating the influence between clusterin proteins and SNPs with other relevant risk factors and interacting proteins would provide a meaningful strategy to unravel mechanistic roles behind AD and other neurodegenerative disorders.

## Conclusion

Clusterin was linked with AD shortly after its identification in 1983. Much of the subsequent focus over the last thirty years has been driven by the discoveries of elevated levels of clusterin in the AD brain, its ability to bind Aβ peptides and the latter discovery of clusterin genetic variants as AD risk factors. Despite these initial leads, insight into clusterin mechanism has been slow to unravel. These early observations have lent weight to the notion that clusterin conferred neuroprotection by acting as an Aβ chaperone leading to clearance of toxic protein fragments. However, it is clear that clusterin is a more complex protein and has both neurodegenerative and neuroprotective functions. Much of this review has concentrated on unraveling this Janus-like character. A large part of the challenge in understanding the role of clusterin in disease arises from the complexity of its biogenesis that results in multiple protein species associated with distinct cellular functions. This inherent complexity is further confounded by frequent lack of clarity and numerous discrepancies in the literature. Nonetheless, a consensus is emerging based on studies of diverse disease states including neurodegeneration, cardiovascular disease and oncology that link secreted clusterin with cytoprotection or anti-apoptosis while intracellular forms mediate apoptosis.

The observation that clusterin levels are increased in several neurodegenerative diseases, all of which share the presence of toxic protein aggregates as a common pathological hallmark, has been used to support the notion that the neuroprotective role of secreted clusterin is likely due to its chaperone function. However, increased clusterin levels are also found in non-proteotoxic neurological diseases, including schizophrenia, Rett syndrome and hypoxia-ischaemia, all of which share at least some underlying pathogenic processes with those found in AD, clearly indicating that the role of secreted clusterin in AD may be more complex than just that of a chaperone. The neuroprotective function of clusterin is evident from studies of *CLU* KO although constitutive KO in mouse leads to both protective and neurodegenerative effects. Given that removal of the entire gene leads to non-discriminative loss of all clusterin forms at all stages of development, such contradictory results are perhaps not so surprising. More nuanced interrogation of the roles of specific protein species will be revealed by using gene editing to control production of specific *CLU* variants and cellular trafficking. We have already seen how these approaches have led to identification of an intronic SNP that acts as an enhancer for clusterin and two neighboring genes, all of which are AD risk genes. Many other SNPs are associated with altered plasma clusterin expression and regulatory functions and gene-editing offers an opportunity to exploit these to identify the mechanisms by which variants influence clusterin structure and function, and LOAD risk. More recently, genetic and biological interactions between clusterin and other AD-relevant genes and proteins such as TREM2, APOE and BIN1, among others, have been discovered. The observation of a link in biological interactions between these proteins is itself interesting, although the exact physiological roles are not clearly defined, but what is more interesting is that genetic mutations in these genes that are themselves associated with AD also influence their interactions. Again, the potential of increasingly sophisticated CRISPR-based technologies to manipulate expression of multiple genes in parallel will allow exploration of gene interactions and identification of gene networks that govern the balance of protection and degeneration. We have already seen how CRISPR/Cas9 KO of clusterin in human iPSC-derived neurons leads to refractoriness to extracellular Aβ and future studies extending these approaches and interrogating function in iPSC-derived neurons, astrocytes and microglia will identify both cell autonomous and non-autonomous actions of the different clusterin species.

We have learned much about clusterin in the last 35 years but as is so often the case, the more we know, the more there is to be known. The very complexity of clusterin function may prove to be beneficial in identifying downstream pathways and searching for potential therapeutic targets that distinguish between its pro-apoptotic and anti-apoptotic actions. Although the role of clusterin has often been examined through the lens of individual disease pathologies, clusterin responds to numerous stressors including inflammation, oxidative stress, ER stress, and proteotoxic stress, all of which underlie a range of pathologies including cancer, cardiovascular disease, and neurodegeneration. There is much to be gleaned from interrogating clusterin function in these disparate disease processes that may be of direct relevance to understanding the role of clusterin in AD and which could be useful for exploiting its therapeutic potential. The growing tool box of gene editing tools, the availability of iPSCs from well characterized patient backgrounds, and increased sophistication in building molecular networks from multi-modal ‘omics’ data will all accelerate this search.

## Bibliography Search and Selection Criteria

Our bibliography search was performed through Europe PMC, and included the terms (“clusterin” or “apolipoprotein j” or “apoj”) and (“Alzheimer” or “neurodegener^∗^”) until August 2018. It was further completed with initial key papers in the discovery and characterization of clusterin gene, protein, and function, as well as papers from other disciplines relevant to the mechanisms explored in this review, found through PubMed search and in the bibliography of selected papers. Selection was based on relevance to the scope of this review.

## Author Contributions

EF and AD-V wrote the draft manuscript. NB, ER, and SL revised and edited the manuscript.

## Conflict of Interest Statement

The authors declare that the research was conducted in the absence of any commercial or financial relationships that could be construed as a potential conflict of interest.

## Footnotes

1 alzgene.org

2 https://www.ncbi.nlm.nih.gov/
